# Russian dilemma for global arctic science

**DOI:** 10.1007/s13280-024-02038-z

**Published:** 2024-06-01

**Authors:** Gareth Rees, Ulf Büntgen

**Affiliations:** 1 Department of Geography, University of Cambridge, Cambridge, CB2 3EN UK; 2 Scott Polar Research Institute, University of Cambridge, Cambridge, CB2 3EN UK; 3 Global Change Research Institute of the Czech Academy of Sciences (CzechGlobe), 603 00 Brno, Czech Republic; 4 Department of Geography, Faculty of Science, Masaryk University, 613 00 Brno, Czech Republic

**Keywords:** Arctic science, Crisis, Russia, Scientific collaboration

## Abstract

Polar regions are critically implicated in our understanding of global climate change. This is particularly the case for the Arctic, where positive feedback loops and climate tipping points enhance complexity and urgency. Half of the Arctic and much of the world’s permafrost zone lie within Russian territory. Heightened geopolitical tensions, however, have severely damaged scientific collaboration between Russia and previously well established academic partners in western countries. Isolation is now causing increasingly large data gaps in arctic research that affect our ability to make accurate predictions of the impact of climate change on natural and societal systems at all scales from local to global. Here, we argue that options to resume both practical knowledge of collaborative working and flows of research data from Russia for global arctic science must continue to be asserted, despite an increasing tendency for the Arctic to become disconnected. Time is short, as preparations for the fifth International Polar Year begin to gather momentum. While sanctions remain in place, efforts to foster peer to peer connections and re-activate effective institutional cooperation are vital to address the grand challenges of global climate change.

## Importance of the Arctic for understanding global climate change

Recognition that the polar regions are essential to our understanding of the Earth’s climate system has become much more widespread over the last couple of decades. They are prominent as both indicators and drivers of climate change. The concept of ‘arctic amplification’ is widely recognised (Rantanen et al. 2022), and eight of the sixteen recently identified climate ‘tipping points’ are geographically located in the Arctic or Subarctic (Armstrong McKay et al. 2022). Of the six most imminent tipping points (in danger of being triggered by a global mean temperature increase well below 2 ˚C), five are within the polar regions, all but one of which are in the Arctic or Subarctic. The behaviour of permafrost is especially implicated in this analysis.

**Fig. 1:**
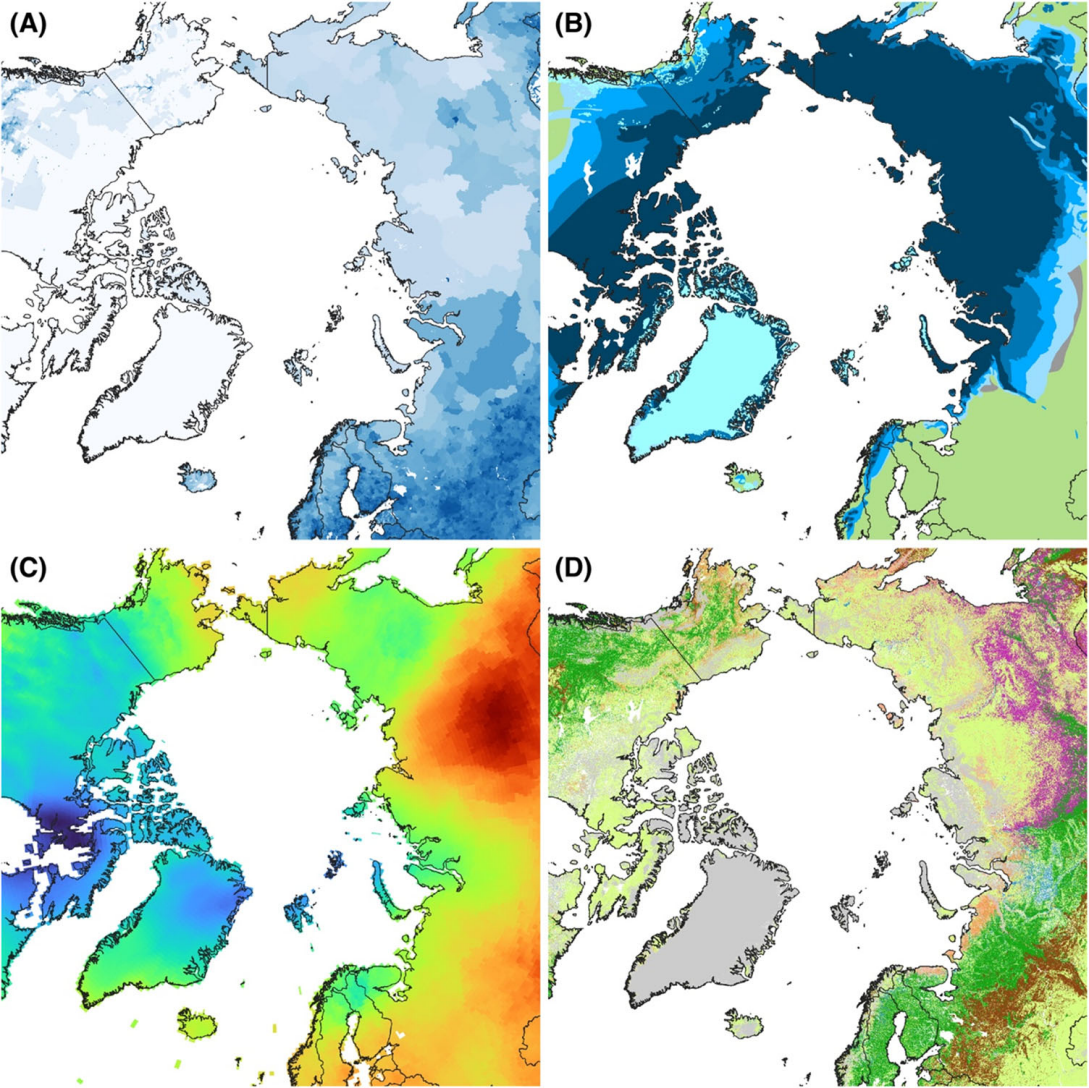
**a** Gridded population density 2020 (GPWv4: https://sedac.ciesin.columbia.edu/data/collection/gpw-v4). **b** Permafrost and ground ice conditions (https://nsidc.org/data/ggd318/versions/2). **c** December to February average temperature trend from 2010 to 2020 (CRU TS data, processed WGR). **d** ESA CCI Plant Functional Type, 2020 (https://catalogue.ceda.ac.uk/uuid/26a0f46c95ee4c29b5c650b129aab788)

Half of the Arctic lies in Russian territory, and there are profound asymmetries between the Russian and non-Russian parts of the Arctic. Amongst other differences, around 60% of the world’s permafrost is in Russian soil (Fig. 1). As such, one cannot fully represent arctic processes without understanding the Russian part of it (Büntgen and Rees 2023). International coordination of arctic science was strengthened by the establishment of the International Arctic Science Committee (IASC) in 1990. This non-governmental organisation included Russian engagement from its outset, and in 1994, it established the International Science Initiative in the Russian Arctic (ISIRA) as an advisory group (Pavlenko et al. 2021). ISIRA had, and continues to have, membership from many countries with arctic research interests, including Russian participation. IASC played a major part in planning and implementing the fourth International Polar Year (IPY) of 2007–2008, and ISIRA helped to guide the equal participation of Russian arctic science within IPY.

International interest in the Arctic has been growing over recent decades, for complex and interlinked reasons that include, but are not limited to, its fundamentally critical role in the global climate system (Huntington et al. 2022). This interest extends well beyond the arctic states. A major strengthening of international collaboration in arctic science was provided by the legally binding Agreement on Enhancing International Arctic Scientific Cooperation, signed into effect at the Arctic Council’s ministerial meeting in 2017.

**Fig. 2:**
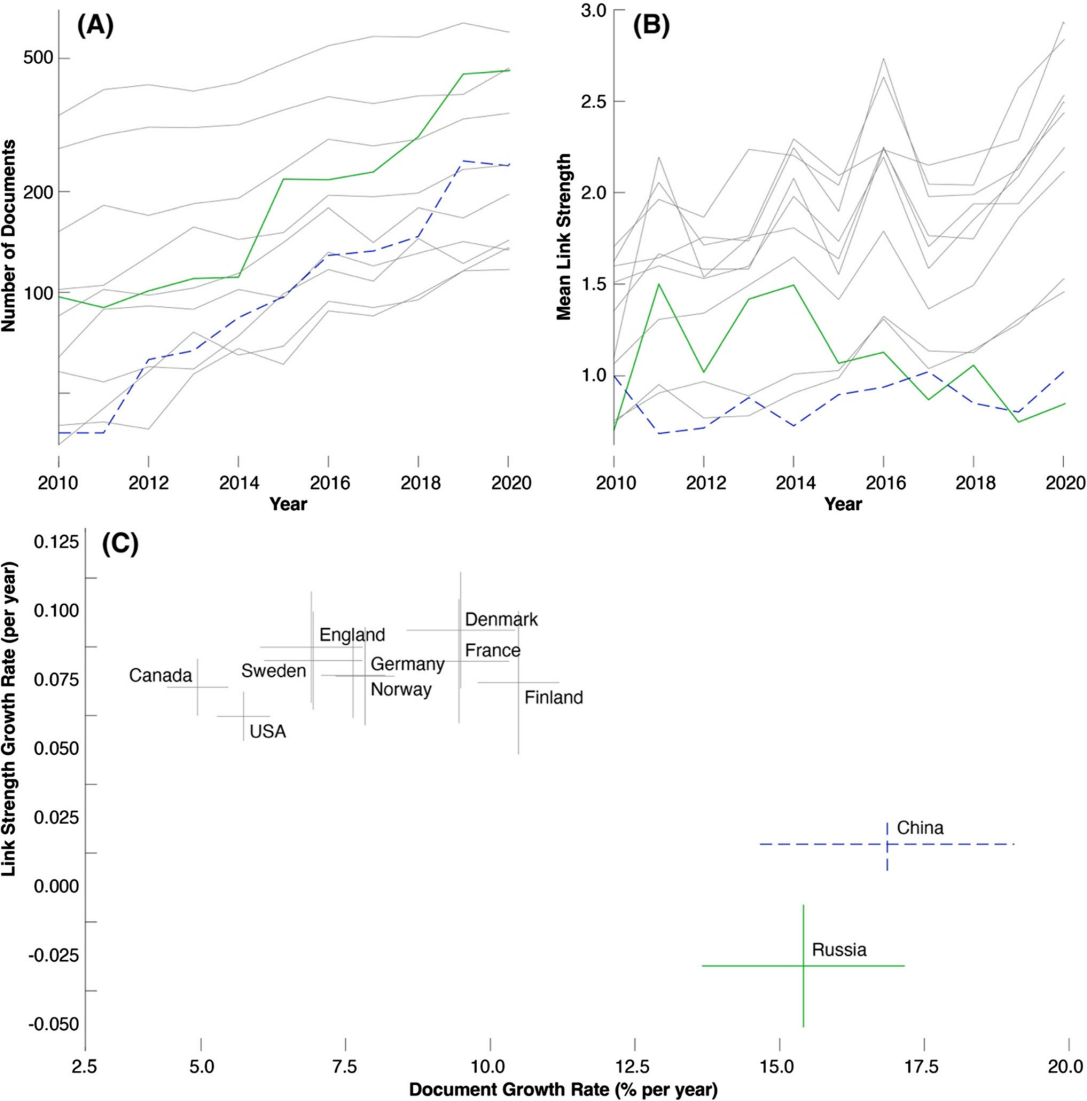
**a** Number of arctic documents published by the 11 most prolific countries between 2010 and 2011. Documents were identified by searching for titles including the word ‘arctic’ in Web of Science in late February 2023. **b** Mean link strength per arctic document for the 11 most prolific countries between 2010 and 2020. Link strength is defined using bibliometric co-authorship analysis in VOS Viewer (https://www.vosviewer.com/): a publication that has co-authors in two countries contributes one link between those countries. Countries with high link strengths thus collaborate internationally more than those with low link strengths. Green and dashed blue lines refer to Russia and China, respectively. **C** Trends in mean link strength per document and number of arctic documents published by the 11 most prolific countries between 2010 and 2020 CE. Results are defined by linear regression analysis, and error bars denote one standard deviation

The first decades of the twenty-first century, however, have been less positive for international engagement in arctic research. While most of the active countries have shown increasing rates of both arctic publication and the international connectedness of their arctic research (Aksnes et al. 2023), this has not been true for two important nations: China and Russia. While both countries have increased their publication rate markedly over the decade since 2010, their degree of international collaboration has remained low and has in fact declined in the case of Russia (Fig. 2).

## Isolation of Russian arctic science from western science

The process of disengagement of Russian arctic—and other—sciences from much of the rest of the world has been abruptly accelerated in the wake of Russia’s full-scale invasion of Ukraine in February 2022, as part of the many consequences of this tragic event (Dodds et al. 2023). Bilateral programmes towards strengthening academic collaboration, in arctic science and elsewhere, active as late as January 2022, were terminated almost instantly.^1^ The subsequent western isolation of Russia’s scholars has unfolded an ever-growing crisis that concerns the Arctic in particular. The weeks immediately following the military invasion were deeply confused, with international organisations unsure how to respond. Global opinion was and remains divided about how to respond to Russia’s aggression, and a coherent strategy towards the Arctic has become difficult to maintain. The Arctic Council, brought into action in 1996 as a consequence of an initiative by Mikhail Gorbachev and chaired at the time of the invasion by Russia, was in effect paralysed, and argued by some to have died. Russian participation in the Arctic Science Summit Week held in Tromsø in March 2022 was non-existent, and no meeting of ISIRA took place that year. Since that time, although chairmanship of the Arctic Council successfully passed from Russia to Norway in May 2023, with some resumption of activity, scientific cooperation in the Arctic has weakened further. Institutional-level collaboration between ‘western’ countries and Russia is generally not permitted, and even though peer-to-peer collaboration is usually tolerated, conditions for it are far from favourable. Economic sanctions imposed on Russia limit researchers’ access to equipment and consumables. The ‘brain drain’ has been huge, with over a million people estimated to have left Russia since the invasion, of whom most are highly educated (Inozemtsev 2023). Of those scientists who remain, it can be presumed that some at least will be afraid of being seen to work with scientists in ‘unfriendly’ countries (Cornwall 2023). Likely facing similar issues, western scientists may also be cautious of jeopardising the safety of their Russian colleagues—and often friends. Trust and openness are rapidly giving way to increasing suspicion and fear, at least between Russian and western scientists.

## Why does all this matter?

The location of sites from which internationally accessible field data have been collected across the Arctic and Subarctic was not uniform even before the recent rise in Russia’s isolation (Metcalfe et al. 2018). Long-term issues included difficulties of data sharing and the use of internationally agreed measurement protocols, especially those related to permafrost monitoring (Bouffard et al. 2021). The probable quantitative impact of excluding data from Russia has been estimated based on the INTERACT network of arctic field stations (López-Blanco et al. 2024), and specifically for arctic carbon flux measurements (Schuur et al. 2024). There is now evidence for substantial decreases in representativeness, with biases in some cases as large as the predicted climate change signal by the end of the twenty-first century. Predictions of change in vegetation biomass are especially divergent. An increasingly unrepresentative spatial distribution of cross-verifiable arctic field data and a diminished flow of long-term monitoring data represent a major data gap (Schuur et al. 2024). On current trends this will only become larger as a consequence of increasing deglobalisation of science, with fewer and more disconnected Russian-based researchers with declining access to suitable measurement tools, and potentially divergent measurement protocols. As already noted, measurements made in more accessible parts of the Arctic cannot straightforwardly be extrapolated into the Russian Arctic and although remote sensing from satellite platforms does give access to many global variables (Pisek et al. 2023), it cannot fully replace on-the-ground data. Some important quantities cannot anywhere be reliably estimated from spaceborne data (e.g. A. Kirdyanov, quoted by Dobrovidova 2023), and others require regionally specific validation if they are to be reliably estimated from satellite data. The uniqueness of complex environments in high northern latitudes of Russia and the preponderance of permafrost there point very clearly to the ongoing need for in situ environmental data collection.

These are arguments that favour the kind of circumpolar cooperation that brought into being IASC, ISIRA and the Arctic Council, but the Arctic is shifting in a different geopolitical direction, increasingly bifurcating into a ‘Russian-Asian’ and a ‘Western’ sphere (Andreeva et al. 2024). It is significant that all seven of the non-Russian arctic states that comprise the membership of the Arctic Council are now members of NATO.

## The urgency to re-integrate Russian arctic science

IASC and the Scientific Committee for Antarctic Research (SCAR) have announced that preparation for the fifth IPY (2032–33) has begun.^2^ An important precursor to the IPY will be the fourth ICARP (International Conference on Arctic Research Planning), in March 2025,^3^ and the ICARP-IV steering committee has convened seven Research Priority Teams (RPTs). These RPTs, already active, will help to identify knowledge gaps, set research priorities, and suggest collaborative partnerships. A diversity of Russian voices needs to resonate in this process.

While western governments rightly continue to isolate Russia for its aggression against Ukraine, top-down intergovernmental and INGO incentives for collaborative Russian arctic science seem unlikely to resume in any meaningful way. International collaboration is not a tap that can be quickly turned on again after having been closed. Networks of understanding, common purpose and trust require time and patience to develop and thrive, though they decay rapidly when not maintained. Russian science is increasing partnerships with China and other countries not classed as ‘unfriendly’, but these will take time to become effective and will not necessarily result in open global networks. It is more important than ever that we find mutual ways to nurture the kind of existing peer-to-peer connections on which trustful collaboration is built sustainably and to encourage new ones to develop. The grand challenges both natural and societal systems are facing in the twenty-first century emphasise the urgency for new links to be developed, even if institutional-level networks must remain dormant for the time being. It seems that the role of science diplomacy in the arctic needs to be expanded urgently. Some mechanisms are operational. Russia is not fully disengaged from international arctic fora, remaining active in research in Svalbard, and still party to the Central Arctic Ocean fisheries agreement. While the Arctic Council and IASC are unable to engage with Russia at the highest level, membership of their working groups allows some interaction to occur. These must be supported and built on if truly international research collaboration in the Arctic is to be restored.

## Footnotes

1 E.g. https://www.uarctic.org/news/2022/1/second-russia-uk-university-rectors-forum-took-place-in-moscow/.

2 https://iasc.info/cooperations/international-polar-year-2032–33/.

3 https://icarp.iasc.info/.
